# Safe and Perilous Occupations

**Published:** 1893-01

**Authors:** 


### Safe and Perilous Occupations.

Some one has facetiously observed that of all occupations that of the assassin is the most conducive to longevity. Certain it is that no sooner is a person known to have committed murder than all the safeguards that human ingenuity can devise are thrown around him and everything possible is done to prolong his days on earth in comfort, ease, and even luxury. If the vast sums of money, the valuable time, the brilliant talent, the profound learning, the resistless energy, and the nauseating sympathy now wasted on murderers were applied to improve the sanitary condition of our schools it would be more humane, and the result would be increased health, wisdom, and morality. There were twenty-nine homicides recorded in this city last year, and the number not recorded probably reached up into the hundreds, for there are many here who live by taking human life, and advertise their bloody trade openly in the newspapers; yet we feel sure that it would be a profitable business venture for any insurance company to issue policies at reduced rates on the lives of this great army of assassins, from the bold and venturesome highwayman to the sneaking and cowardly abortionist.

Among the learned professions, that of pointing the way to heaven keeps its votaries longest on earth, while those who engage in holding others back, or smoothing their path, if go they must, glide swiftly on themselves, and soon lose their feeble grip on worldly things; thus, according to English statistics, the death-rate among physicians, between 25 and 65 years of age, is more than twice that of clergymen of the same age, lawyers keeping about equally distant in the race for immortality between those who preach and those who practice. Of course, it is easy to see why medical men die young: irregular habits, loss of food and sleep, exposure to the extremes of weather, jolting and shaking over rough roads, inhaling microbe-laden dust or the foul air of a close carriage, the constant mental strain of weighing diagnostic symptoms and therapeutic indications with the fear of erring where human lives are at stake, and, last but not least, alas! with many of us, the worry expressly forbidden by the

Master according to St. Matthew 6: 25–34—all these various agencies speed our journey.

Of manual toilers, those whose occupation keeps them outdoors are, with some few exceptions, the longest-lived, the exceptions being due to other causes, as overwork, especially sudden muscular efforts and strains, liability to accident, and exposure to inhalation of dust and poisonous vapors. Thus, gardeners, farmers and fishermen are exceptionally long-lived, and sailors would be so but for the poor quality of food, insufficient and frequently bad water, and the cramped-up, damp, dingy sleeping quarters furnished them. In these respects there has been a great improvement of late years, but much remains yet to be done. Jack's riotous living ashore, and too often insufficient clothing at sea are also responsible for many of his ailments.

Carpenters and masons, whose work keeps them mostly in cities, where the air is less pure than in the country or at sea, are not as healthy as farmers or fishermen, and painters and plumbers, who are almost constantly exposed to noxious vapors, and suffer more or less at all times from chronic poisoning, die comparatively young.

Tailors and shoemakers, who not only live in a foul atmosphere, but also sit all day in such a cramped position that respiration and digestion are interfered with, as well as drapers, wool and cotton workers, cutlers, file-makers, and printers, are liable to phthisis.

Liquor sellers and hotel waiters are extremely short-lived, their death-rate being respectively two and three quarters and four times as great as that of clergymen.

Railroading and other occupations, requiring one to be more or less constantly on the road, are extra hazardous, not so much because of the accidents to which one is exposed, as because of the continued jarring, the superheated and foul air in the car and severe draughts every time a door or window is opened, and the fine dust which settles not only in the air passages, but almost completely clogs up the pores of the skin, throwing extra work on the kidneys and giving rise to the so-called "railroad kidney." For this reason as well as for the broken sleep and irregular meals, commercial travellers are undesirable life-insurance risks.

To be a capitalist, whether busy or idle, is somewhat risky; for, besides being a target for dynamite bomb-throwers, if busy, the physical wear and tear and the mental strain and anxiety of speculations will soon shatter both your mind and body, consigning you either to the madhouse or a premature grave, and, if idle, dissipation or *ennui* are apt to finish you early.

This being a general election year we should fail in our duty to the public if we did not remind our readers that they may also in their official capacity warn the people of the dangers to which the politician is exposed. On this subject we can not do better than to quote an editorial in the *Medical Press and Circular*, June 22d, 1892:

"The excitement associated with an approaching general election possesses a distinctly medical interest, as practitioners all over the country will shortly have another opportunity of ascertaining for themselves. Apart from the surgical injuries and solutions of cutaneous continuity caused by the impact of brickbats and missiles of a similar description, to be treated *sec. art.*, the excitement and the exhausting physical exertions which canvassing and electioneering entail upon the candidate and his chief agents determine tangible proportion of breakdowns. It has often been noticed that the election is barely over before a certain number of the candidates collapse and are forced to retire from active political life. Indeed, one is surprised that life assurance companies do not insert into the conditions of the grant of the policy a saving clause relieving them from all responsibility during the electoral period. Given a mature age and a sturdy determination to succeed, the position of a parliamentary candidate certainly falls within the category of dangerous occupations. The wonder, indeed, is that a larger number do not give way under the strain, but the effects can not be measured by the immediate mortality. The moment seems opportune to advocate the value of blood-letting in heart failure. Such an operation, carried out on a public platform with promptitude and despatch on a syncopal chairman or lecturer, would be enough to secure a popular reputation for the operator, especially if by good luck the victim survived the ordeal."

Several defeated Presidential candidates have lain down and died shortly after their defeat, and Generals Garfield and Arthur might have been alive to-day had they left politics alone.

From what we have said it follows that if you would enjoy a happy life, as well as a long one, and be prepared to go to a better place when your time comes, practice not, but rather preach; spend not your days in houses built by man, but under God's fair sky; seek not wealth, for "it is easier for a camel to go through the eye of a needle than for a rich man to enter the kingdom of heaven;" keep the ten commandments and read and *heed* daily the divine injunction: "Take no thought for your life, what ye shall eat or what ye shall drink; nor yet for your body, what ye shall put on."—*Pacific Medical Journal*, August 1892.

